# Artemisinin and Derivatives-Based Hybrid Compounds: Promising Therapeutics for the Treatment of Cancer and Malaria

**DOI:** 10.3390/molecules26247521

**Published:** 2021-12-11

**Authors:** Sijongesonke Peter, Siphesihle Jama, Sibusiso Alven, Blessing A. Aderibigbe

**Affiliations:** Department of Chemistry, University of Fort Hare, Alice 5700, South Africa; 201414787@ufh.ac.za (S.P.); 201406239@ufh.ac.za (S.J.); 201214199@ufh.ac.za (S.A.)

**Keywords:** cancer, malaria, artemisinin, combination therapy, hybrid compounds, multidrug resistance

## Abstract

Cancer and malaria are major health conditions around the world despite many strategies and therapeutics available for their treatment. The most used strategy for the treatment of these diseases is the administration of therapeutic drugs, which suffer from several shortcomings. Some of the pharmacological limitations associated with these drugs are multi-drug resistance, drug toxicity, poor biocompatibility and bioavailability, and poor water solubility. The currently ongoing preclinical studies have demonstrated that combination therapy is a potent approach that can overcome some of the aforementioned limitations. Artemisinin and its derivatives have been reported to exhibit potent efficacy as anticancer and antimalarial agents. This review reports hybrid compounds containing artemisinin scaffolds and their derivatives with promising therapeutic effects for the treatment of cancer and malaria.

## 1. Introduction

Malaria cases are increasing globally and this negatively affects communities because of the socio-economic and health burden associated with this parasitic disease [1]. There are four well-known plasmodium parasites—namely, *P. falciparum, P. vivax, P. ovale,* and *P. malariae.* The recently reported plasmodium parasite, *P. knowlesi* also causes human malaria [2,3,4,5]. The most deadly is *P. falciparum*, followed by *P. vivax* in some regions of the world [1]. Malaria is an infectious, chronic, and deadly disease in humans. Several research approaches have been designed to combat this disease. However, these approaches have been hampered seriously by factors such as drug resistance, toxicity, regions with different dominant malaria species, malaria vectors, source of the disease, etc. [3,4].

African and Asian countries report high cases of malaria deaths and there is a WHO plan to eradicate malaria in the next decade [1]. However, this process is challenging because evaluating the vector and human behaviour is the first approach to develop effective therapies to eliminate malaria [6,7,8]. In the last century, malaria caused serious problems with over 77% of the world population suffering from the disease, but the efforts to eliminate malaria were regarded as successful because malaria in the affected population decreased from 77% to 48% over the last 100 years, and quinoline antimalarial drugs such as chloroquine, mefloquine, and quinine have been indicated as drugs responsible for the successful control of malaria [9]. The currently used antimalarial drugs are experiencing some drawbacks such as drug resistance, toxicity, etc., and over 450,000 people die yearly because of this disease, worldwide [9,10,11]. Another chronic disease that results in a high rate of morbidity and mortality around the world is cancer. 

**Figure 1:**
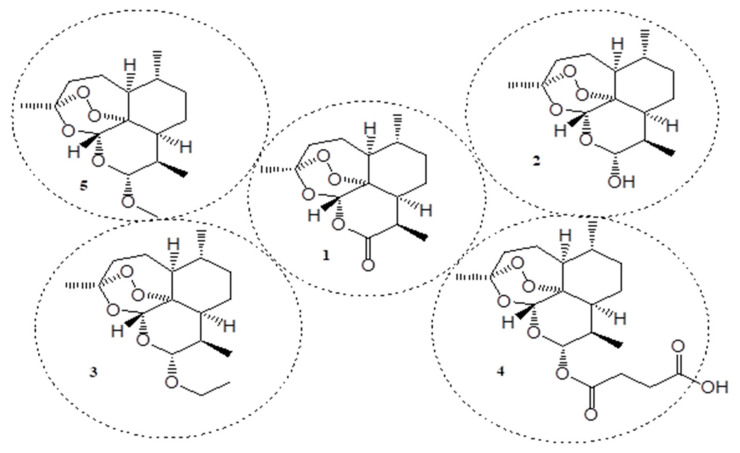
Chemical structures of artemisinin and some of its derivatives (**1**–**5**).

Cancer is also a chronic disease, and its burden continues to grow globally, disrupting health systems, families, communities, etc. In 2018, 18 million new cases and 9.6 million cancer-related deaths were reported. An increase of 1.2 million cancer cases and 400,000 deaths between 2018 and 2020 were estimated using the GLOBACAN database in 2021. Over 5–10% of cancer cases were reported to be via inherited genes while 90–95% of cancers cases were believed to be caused by gene mutation [12,13,14]. The currently used anticancer agents suffer from drug resistance and poor specificity, resulting in some adverse side effects. Therefore, there is a pressing need to design and develop new and effective anticancer agents [12]. In the process of discovering novel therapeutic agents for the treatment of cancer and malaria, artemisinin (**1**) has been discovered. Artemisinin (**1**) (qinghaosu) is a compound extracted from a medicinal plant found in China known as *artemisia annua*. It has been used in China for over 100 years to treat fever and chills [15]. The combination of artemisinin with other bioactive agents is one potent strategy to overcome the challenges faced by known anticancer and antimalarial drugs. Artemisinin is currently one of the first-line treatments for malaria and it is prominent in the medicinal industry because it exhibits a wide range of biological activities including antimalarial, antifungal, anticancer, anti-HIV, antibacterial, etc. The presence of its unique peroxide bridge has been reported to be the reason for its diverse biological activities [16,17]. In 2006, Artemisinin Combination Therapy (ACT) was reported as one of the most effective therapy to treat chloroquine-resistant *P. falciparum* and *P. vivax* infections [18,19]. However, artemisinin has a short half-life, limited bioavailability, and poor solubility, and these shortcomings shorten the duration of its therapeutic efficacy [12,19]. A synthetic approach involving the removal of the carbonyl group to improve artemisinin resulted in dihydroartemisinin (**2**) derivative, which when modified through synthesis, led to artemisinin derivatives (Figure 1) (arteether (**3**), water-soluble artesunate (**4**), and oil-soluble artemether (**5**)) with enhanced biological activity [19]. The modification of artemisinin or its use in combination with other therapeutics improves its anticancer or antimalarial activity. In this review, we report the in-vitro and in-vivo outcomes of hybrid compounds containing artemisinin scaffolds and their derivatives.

## 2. Mechanisms of Artemisinin

### 2.1. Mechanism of Artemisinin in Cancer

Although artemisinin and its derivatives are well-known as antiplasmodial agents, they also demonstrate significant cytotoxic effects on many cancer cell lines including lung, breast, gastric, liver, colon, leukaemia, cervical, melanoma, osteosarcoma cells, in vitro [20]. There are many proposed mechanisms of action of artemisinin and derivatives on cancer cells, but they are all dependent on the molecule’s ability to inhibit cell growth or destroy the cell cycle stages via proliferation pathways [21,22]. Some studies have revealed that the endo-peroxide bridge of artemisinin interacts with either the intracellular iron or heme groups resulting in the production of radicals that are cytotoxic with an alkylating activity [23,24]. An increase in the concentration of intracellular iron can result in increased cytotoxicity of artemisinin by 100-fold if tumour cells are encapsulated with iron or iron-saturated holo-transferrin [24]. Tumour cells display an increased demand for iron, and their iron metabolism rate and the expression of transferrin receptors is higher than the normal cells, causing them to be more susceptible to the cytotoxicity of artemisinin [25]. 

**Figure 2:**
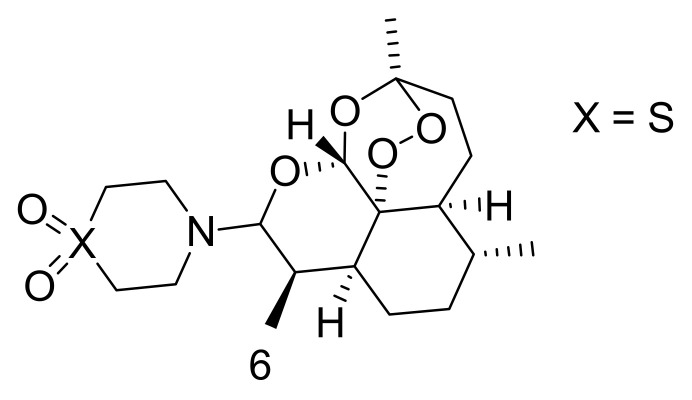
Chemical structure of artemisone (**6**).

Some research reports have revealed low doses of artesunate stimulate oncosis-like cell damage, considered by cytoplasmic vacuolization and swelling, dilation of the nuclei, disorganized mitochondria, and cell lysis. Nevertheless, at higher dosages, it promotes cell apoptosis [26]. Artemisinin can cause cell disruption by accumulating inside the mitochondria and lysosomes, in vitro [27]. The proliferation pathways of cancer cells that can be inhibited by artemisinin include adenosine monophosphate (AMP), Wnt beta signalling pathway (Wnt/Beta) catenin, and second messengers involved in intracellular signalling (nuclear factor kappa-light-chain-enhancer of activated B cells (NF κB), CREB binding protein (CREBBP), MYC, angiogenesis factors, as well as mechanistic target of rapamycin (mTOR) [28]. Hooft van Huijsduijnen et al. reported that dihydroartemisinin, artemisinin, and artemisone (6) (Figure 2) stimulate apoptosis via the intrinsic pathway involving caspase-9 and caspase-3, while other studies showed artemisinin capability to promote apoptosis in vitro in murine mastocytoma (P815) cells [29].

Another mechanism of artemisinin is via inhibition of metastasis [30]. Weifeng et al. demonstrated that artemisinin prevents metastasis by increasing cell to cell attachment through enhanced expression of Cdc42 and increased efficacy of the E-cadherin protein [31]. All the reported mode of actions of artemisinin and its derivatives reveals their anticancer activities through pleiotropic effects, including inhibiting the proliferation of cancer cells, inducing apoptosis, stimulating cell cycle inhibition, inhibiting angiogenesis, mediating the tumour-associated signaling pathways, destroying cancer metastasis and invasion, and regulating tumour microenvironment [32,33]. More significantly, artemisinin and its derivatives exhibit minor toxic effects on normal body cells and overcome multidrug resistance that is commonly reported in most anticancer drugs [24].

### 2.2. Mechanism of Artemisinin in Malaria 

Artemisinin mode of action is not still fully understood but the presence of the endoperoxide bond is responsible for its potent biological activity [34,35]. It is selective towards erythrocytes infected with parasites when compared to the uninfected erythrocytes and it is also influenced by the iron-dependent bioactivation of the endoperoxide bridge [35]. During parasite haemoglobin degradation by the protease enzymes, peptides and amino acids are released to create space within the digestive vacuole and used for parasite growth; however, this process results in haematin formation which is toxic to the *P. falciparum* parasite [35]. The parasite develops a mechanism (whereby the hematin undergoes biomineralization) that can overcome this toxicity by forming haemozoin which is non-toxic. Highly reactive oxygen radicals formed from the cleavage of the peroxide bridge in the presence of ferrous ion (Fe^2+^) from heme are quickly repositioned to form more steady carbon-centred radicals which are very important for antimalarial activity. It has been proposed that these free artemisinin-radicals results in *P. falciparum* parasite’s death by chemical modification and hinders a variety of parasite molecules [34,35]. 

The development of resistance by *Plasmodium* parasites to artemisinin is due to gene mutation. *P. falciparum* cultures and *P. yoelli* mouse models were reported to develop resistance to artemisinin. The in vitro studies suggest that artemisinin and its derivatives resistance could be multigenic and share similar characteristics with the quinoline class [33,35]. Pharmacological factors such as metabolism are linked with recrudescence after monotherapy with artemisinin drugs. The artemisinin resistance has also been attributed to its short half-life since more time is needed to eradicate the parasite [34,36,37].

## 3. Artemisinin-Based Hybrid Compounds for Cancer Therapy 

**Figure 3:**
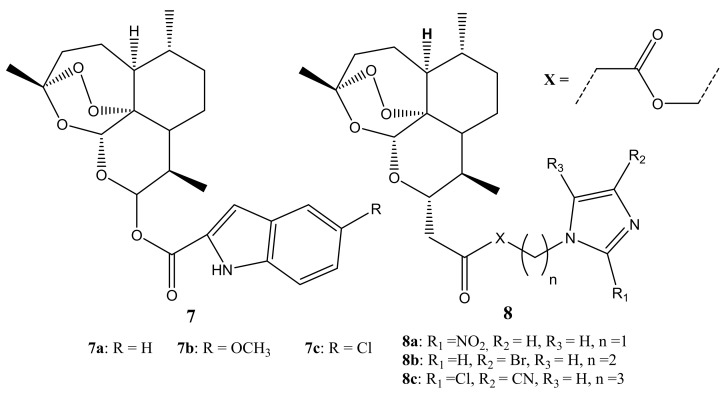
DHA-indole and ATS-imidazole hybrid compounds (**7**,**8**).

There are many reported hybrid compounds containing artemisinin scaffold and its derivatives as promising compounds for the treatment of cancer. Hu et al. synthesized dihydroartemisinin (DHA)-indole **7** and artesunic acid (ATS)-imidazole **8** hybrid compounds (Figure 3) with broad anticancer activity against several cancer cell lines [38]. The hybrid compounds were evaluated in vitro for their cytotoxic effects against non-small-cell lung cancer cell line (A549), human hepatocellular carcinoma cell line (HepG-2), human breast cancer cell line (MCF-7), and human normal liver cell line (L02). Most of the compounds exhibited superior anticancer activity when compared to artesunic acid, dihydroartemisinin, and artemisinin. Specifically, hybrids **7a** and **7c** displayed high anticancer activity against the three cell lines (HepG2, A549, and MCF-7), and **30c** demonstrated cytotoxic effect against MCF-7 with an inhibitory concentration (IC_50_) of 5.25 μM, which was 10-fold more than artemisinin (IC_50_ value of 38.71 ± 5.735.25 μM on MCF-7). These hybrid compounds demonstrated low cytotoxic effects on the L02 cell lines, demonstrating good biocompatibility in normal cell lines [38]. The length of the linker displayed no effect or improvement on the cytotoxicity activity of hybrids **8** [38]. These compounds were further evaluated for their multidrug resistance (MDR) reversal activity against MCF-7/ADR cells since artemisinin suffers from drug resistance. Compound **7a**–**c** exhibited MDR reversal activity which was better than ATS-imidazole hybrids **8a**–**c** with **7c** displaying outstanding results. Moreover, among compounds **8a**–**c**, 2-nitro substitution resulted in less MDR reversal activity than a 4-nitro substitution of imidazole group with cyano or bromine substitution displaying the worst MDR reversal activity. The compounds with fewer carbons in the linker exhibited significant anticancer activity in vitro than compounds with many carbons in the linker [38]. Thus, the position of the substitution and the length of the linker influenced the therapeutic efficacy of these compounds. 

**Figure 4:**
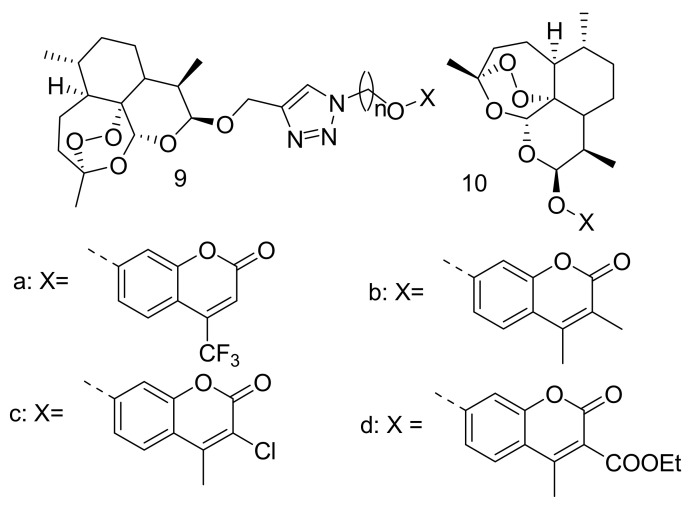
DHA-coumarin hybrid compounds (**9**,**10**).

Tian et al. synthesized dihydroartemisinin-coumarin hybrids (Figure 4) via click chemistry using linkers with different lengths for cancer therapy. The cytotoxic activity of all the hybrid compounds using MTT assay on MDA-MB-231, HCT-116, MRC-5, and HT-29, demonstrated moderate efficacy with IC_50_ values that ranged between 0.05–125.40 µM. These hybrids displayed superior efficacy against HT-29 cell line under the anoxic condition with cytotoxicity which was 10-fold more under anoxic condition than normoxic condition [39]. Among the synthesized derivatives, compounds **9a**–**d** displayed good selectivity against the HT-29 cell line. The length of the linker and the functional groups present on the coumarin moiety affected the anticancer activity of these compounds [39]. Compound **9d** was the most potent compound with IC_50_ values of 0.05 µM against HT-29 cell line under the anoxic condition and further studies on the anticancer activity of these compounds are recommended [39]. Further studies were done by Yu et al. by synthesizing a series of artemisinin-based hybrids (**10a**–**d**), containing DHA and coumarin (Figure 4) as potential anticancer drugs [40]. The anticancer activities of the hybrid compounds were higher against human colorectal cancer cell line (HT-29) cancer cells than the human breast cancer cell lines (MDA-MB-231). Further experiments demonstrated that these compounds arrested the G0/G1 phase of HT-29 cells, inhibited the proliferation of HT-29 cell lines, suppressed the migration of cancer cells, and promoted a decrease in mitochondrial membrane potential, resulting in cancer cell apoptosis. These compounds also stimulated the other cell death pathway known as ferroptosis [40]. These compounds exhibited moderate cytotoxicity on both human cancer cell lines, although they differ since they consist of different linkers and functional groups substituted on different positions of coumarin moiety [40]. 

Generally, the observations from these synthesized compounds were that compounds with longer chain length in the linker exhibited better cytotoxic effects than compounds with shorter chain length in the linker, meaning an increase in the chain length of the linker improved the cytotoxicity of these derivatives. In contrast, the length of the linker had no significant effect on the compounds containing 1, 2, 3- triazole linker which exhibited better activity than other compounds from the same series [39,40]. The type of functional groups and the position of the substituents influenced the cytotoxicity of these series of compounds. All the compounds with 3-chloro, 4-methyl substituent displayed superior activity and compounds with 3-methoxycarbonyl or 3-ethoxycarbonyl groups on position 3 of coumarin moiety displayed poor cytotoxicity [39,40]. 

**Figure 5:**
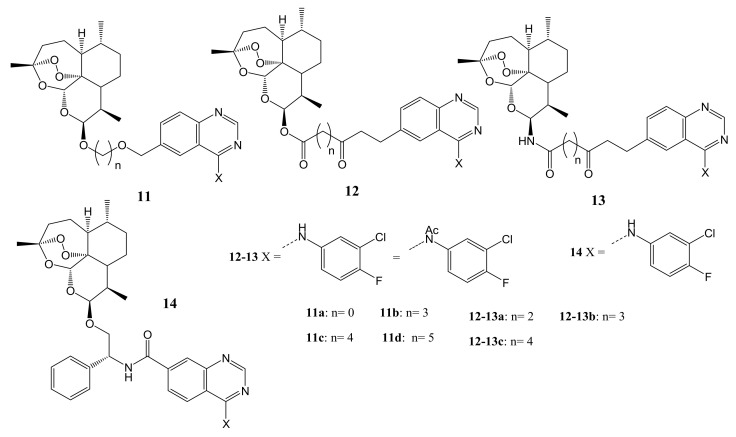
Molecular structures of DHA-4-(arylamino) quinazoline hybrid compounds (**11**–**14**).

Wang et al. synthesized a series of hybrid compounds of DHA-4-(arylamino) quinazoline (**11**–**14**) using ether, ester, and chiral linkers for colorectal cancer therapy [41]. The structures of the hybrid compounds are shown in Figure 5. Compound **11d** demonstrated promising antiproliferative activity with the lowest IC_50_ value of about 0.24 µM which was 10-fold more active than the parent drugs, DHA with an IC_50_ of 2.85 µM. Hybrid **14** with the chiral linker was the most potent compound against colorectal cancer cell lines (HCT116 cell) with an IC_50_ of 110 nM, when compared to the parental drugs, DHA (IC_50_ = 2.85 mM) and gefitinib (IC_50_ = 19.82 mM). The in-vivo studies using HCT116 xenografts revealed that hybrid **14** displayed superior anticancer efficacy with tumour growth inhibition and the tumour shrank after 18 days of treatment on the mice without significant weight loss [41]. For the compounds with ether linkers, the stereochemistry on the anomeric carbon influenced their anticancer activity. The ester and amide bond in the linker also influenced the anticancer activity of compounds. Compounds with di-ester linkers exhibited superior anticancer activity than compounds with amide-ester linkers [41]. 

**Figure 6:**
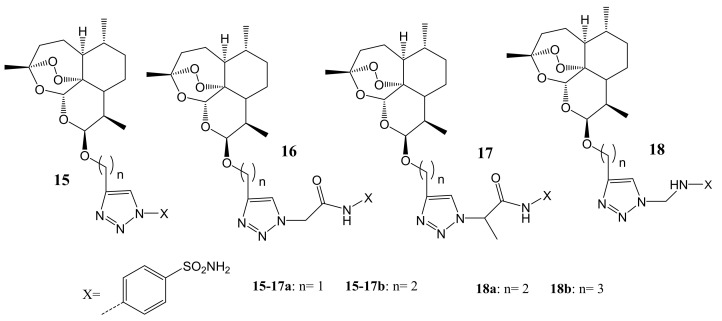
Artemisinin-sulfonamide hybrid compounds (**15**–**18**).

An et al. formulated artemisinin-sulfonamide hybrid compounds (**15**–**18**) (Figure 6) [42]. They evaluated their inhibitory activity against four different isoforms (i.e hCA I, II, IX, and XII) with acetazolamide (AAZ) used as a standard inhibitor. Additionally, the in vitro anticancer analysis was performed using an MTT assay against two cancer cell lines (HT-29 and MDA-MB-231) and the in vitro cytotoxicity was performed using normal human cancer cell lines (MCF-10A) [42]. Among all the synthesized compounds, it was observed that hybrid **15b** with IC_50_ values of about 0.65 μM against HT-29 cell line was the most cytotoxic under hypoxia conditions, and hybrid **18a** exhibited the lowest index (IC_50_ (Hypoxia)/IC_50_ (Normoxia) = 0.057, 0.075. It was further observed that the inhibitory activity trend differs by the isoforms used. However, compounds **15a**, **15b**, **16a**, **18a** and **18b** showed higher inhibitory activity against HT-29 and MDA-MB-231 cancer cell lines in vitro compared with DHA. The in-vitro cytotoxicity studies on p-aminobenzenesulfonamide hybrids **15b**, **16a**, **16b**, **17b** and **18a** revealed no obvious cytotoxic effect on the human normal cell line, MCF-10A cell line (IC_50_ = 52–85 μM) [42]. The position of the substitution is paramount in the development of novel artemisinin anticancer hybrid compounds as p-amino-benzenesulfonamide derivatives were the most active anticancer hybrids than the m-aminobenzenesulfonamide derivatives [42].

**Figure 7:**
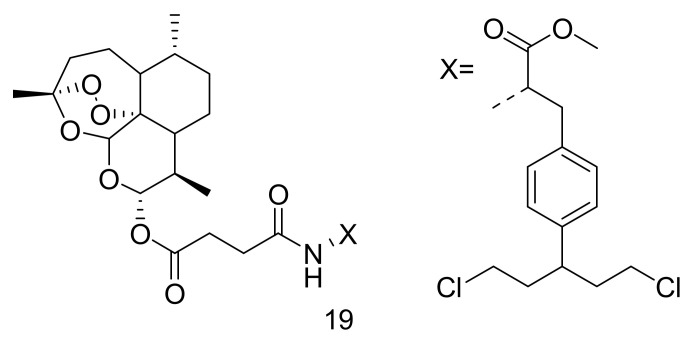
DHA-melphalan hybrid compound (**19**).

Li et al. synthesized artemisinin-based hybrid compounds by bonding melphalan, aminoglutethimide, chlorambucil, doxifluridine, and flutamide at position C-10 of DHA for ovarian cancer treatment [43]. The most effective compound, **19** against ovarian cancer cell lines (A2780 and OVCAR3) was the DHA-melphalan hybrid compound (Figure 7) with IC_50_ values of 0.86 μM and 0.83 μM, respectively, compared with parental drugs, DHA and melphalan. DHA-melphalan hybrid significantly hindered cancer cell growth in a concentration-dependent phenomenon, indicating 67.1% and 59.2% inhibition at 1 μM for OVCAR3 and A2780 cells, respectively. The DHA-melphalan hybrid compound displayed a less cytotoxic effect when immersed with normal ovarian epithelial cells for 2 days with an IC_50_ value of approximately 43.64 μM, suggesting that DHA-melphalan selectively destroyed cancer cells [43]. 

**Figure 8:**
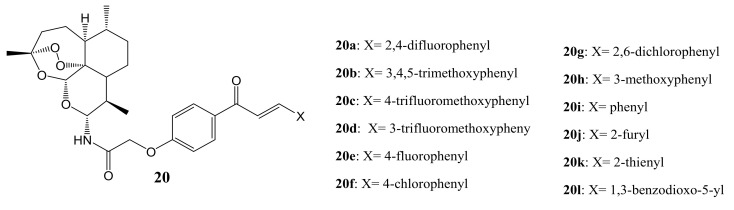
Artemisinin-chalcone hybrid compounds (**20a**–**l**).

Xie et al. designed a series of artemisinin-chalcone hybrid compounds (Figure 8) and evaluated their anticancer activity in vitro against HT-29, A549, MDA-MB-231, human cervical carcinoma cell line (HeLa), and human lung cancer cell line (H460) cell lines using MTT assay and DHA as the control [44]. Most of the hybrids **20a**–**l** were 40-fold more effective than DHA, indicating that the incorporation of a chalcone pharmacophore significantly improved their anti-cancer activity on all the cancer cell lines used in the study. Generally, most of the synthesized hybrid compounds exhibited moderate anticancer activity against A549, MDA-MB-231, and H460 cell lines with IC_50_ values ranging between 0.68 and 29 mM, which was several- to ten-fold less potent than against HT-29 and HeLa cancer cell lines, respectively [44]. However, compounds **20c**, **20d** and **20g** exhibited better anticancer activity against all the cancer cell lines, indicating that the position of substitution (in this case C-10) and introduction of electron-withdrawing and bulkier groups into the phenyl ring were detrimental to the anticancer activity of some of the synthesized compounds, resulting in moderate anticancer activity [44].

**Figure 9:**
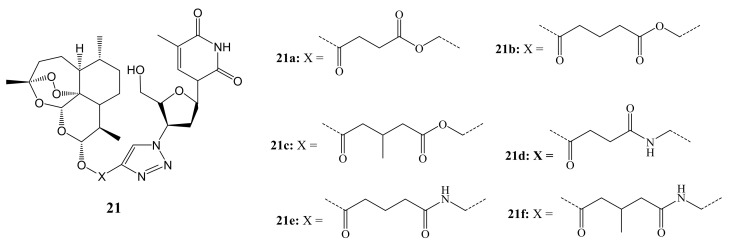
Artesunate-triazole-3′-azido-3′-deoxythydimine (AZT) hybrid compounds (**21a**–**f**).

Tien et al. synthesized artesunate-triazole-3′-azido-3′-deoxythydimine (AZT) hybrid compounds (Figure 9) via “click” chemistry. The in-vitro cytotoxicity evaluations demonstrated that most of the hybrid compounds possessed good anticancer activity against KB and HepG2 cancer cell lines. The hybrids with ester linkers (**21a**–**c**) showed higher anticancer activity with IC_50_ values ranging between 16.5 and 71.4 µM when compared to those with amide linkers (**21d**–**f**) that possessed IC_50_ values ranging between 135 and 178 µM [45].

**Figure 10:**
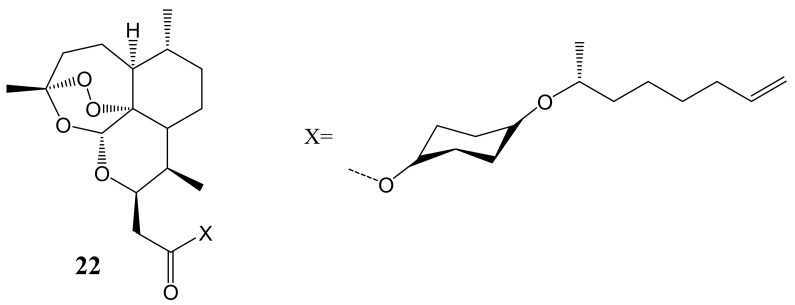
Artemisinin-daumone hybrid compound (**22**).

The artemisinin-daumone hybrid compound (**22**) (Figure 10) reported by Ma et al. demonstrated that the oral administration of hybrid compounds for 6 weeks inhibited the induction of severe osteolytic lesions in the tibiae of MDA-MB-231 breast cancer cell-injected mice in a dose-dependent manner. Artemisinin-daumone hybrids administered at lower concentrations displayed time- and dose-dependent reduction of the cell viability of MDA-MB-231 and A549 lung cells, whereas the administration of artemisinin at a concentration of 80 mM did not induce a significant cytotoxic effect [46].

**Figure 11:**
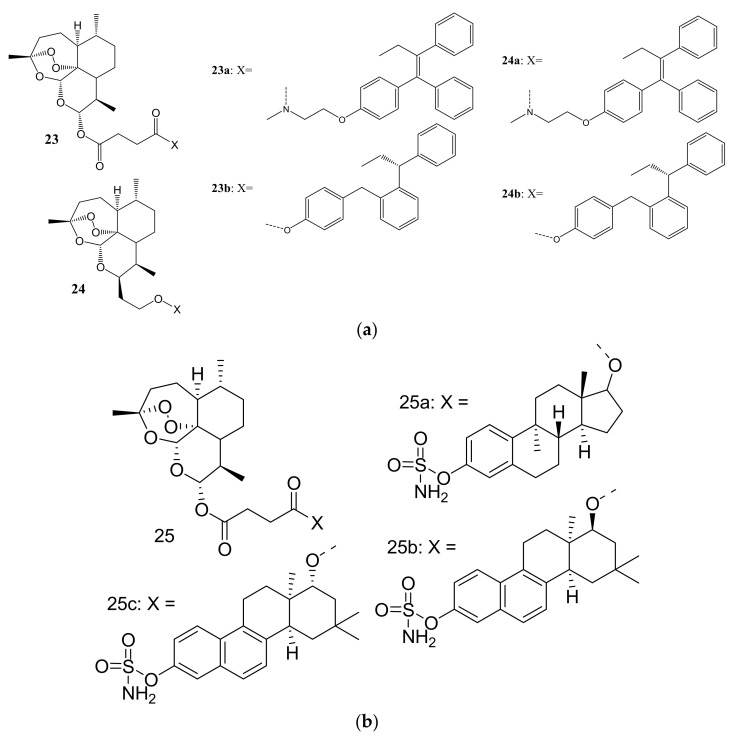
(**a**): Tamoxifen-artemisinin hybrids (**23,24**). (**b**): Estrogen-artemisinin hybrid compounds (**25a**–**c**).

Fröhlich et al. synthesized a library of tamoxifen-artemisinin (**23**–**24**) and estrogen-artemisinin hybrids (**25a**–**p**) (Figure 11a,b) for the treatment of MCF-7 and prostate cancer (PC-3). The biological studies showed that most of the synthesized hybrids possessed enhanced anticancer activity against MCF-7 and PC-3 cancer cell lines than their parent drugs, DHA, artesunic acid, tamoxifen, and estrogen [47]. The promising results from tamoxifen-artemisinin derivatives (**23,24**) resulted in Fröhlich et al. synthesizing estrogen-artemisinin hybrids (**25a**–**c**). Most of the synthesized compounds including tamoxifen-artemisinin (**23,24)** and estrogen-artemisinin hybrids (**25a**–**c**) exhibited enhanced anticancer activity which was superior to that of parent drugs with EC_50_ values ranging between 1.07- 45.6 μM, respectively [47]. Compound **25a** (EC_50_ = 2.08 μM) exhibited a significant activity on MCF-7 cancer cell lines and compounds **25b** (EC_50_ = 1.18) μM and **25c** (EC_50_ = 1.07 μM) both containing a naphthalene moiety displayed a remarkable anticancer activity against PC-3 cancer cell lines, in vitro [47]. The amino groups on these potent compounds were not modified or altered and may have contributed to the enhanced anticancer activity of these compounds [47]. 

**Figure 12:**
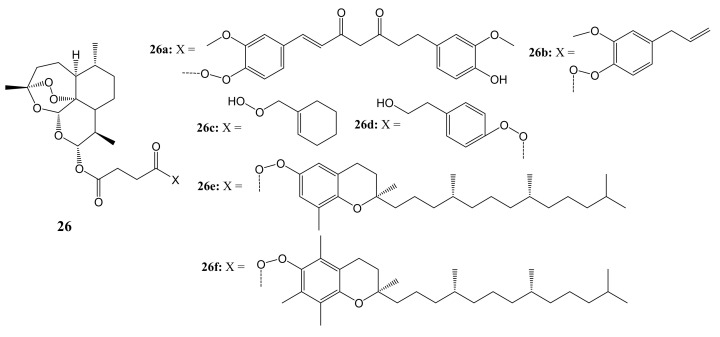
Artemisinin-based hybrids (**26a**–**f**).

Botta et al. synthesized and evaluated artemisinin-based hybrid compounds and dimers for cancer therapy [48]. The structures of the hybrids and dimers are shown in Figure 12. The in-vitro cytotoxicity analysis was performed on HeLa cancer cell lines and three metastatic melanoma cancer cell lines: RPMI-7951, SK-MEL24, and SK-MEL3.

**Figure 13:**
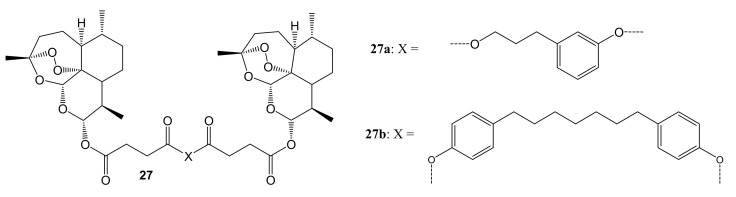
Artemisinin-based dimers (**27a**–**b**).

Artemisinin and its novel artemisinin hybrids **26a**–**f** were found to be selectively cytotoxic towards cancer cells than the normal cell line, C3PV. Moreover, hybrid **26d** displayed high selectivity towards melanoma cancer cell lines but were inactive against HeLa. The dimer **27a** (shown in Figure 13) containing the tyrosol scaffold, exhibited a remarkable antitumor efficacy against both RPMI-7951 and SK-MEL24 melanoma cell lines, with IC_50_ values of 0.24 and 0.49 μM, respectively, which were lower than those of artemisinin and paclitaxel [48]. The toxicity of these artemisinin compounds on cancer cells was influenced by the nature of the phytochemical used in the hybridization [48].

**Figure 14:**
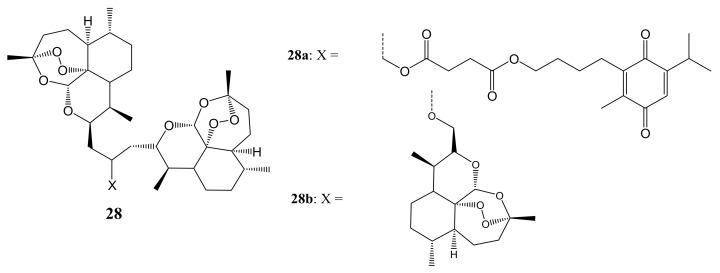
Artesunic acid-based hybrid compounds (**28a**,**b**).

Gruber et al. reported the cytotoxicity effect of artesunic acid-based hybrid compounds (Figure 14). Most of the compounds significantly inhibited cell proliferation by 50% at concentrations below 10 µM except **28a** and **28b**. CEM/ADR5000 cancer cell lines displayed no or negligible cross-resistance towards the compounds. Except for **28a** and **28b**, the degree of resistance of the CEM/ADR5000 cells was much lower than doxorubicin [49].

**Figure 15:**
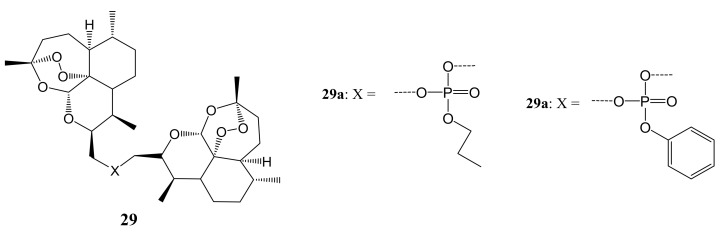
Artemisinin dimers hybrid compounds (**29a**,**b**).

Zhang et al. synthesized a series of novel ring-contracted artemisinin dimers (Figure 15) that possessed anticancer activities. The in-vitro anticancer experiments were performed against six cancer cell lines using an MTT assay. Most of the artemisinin dimers displayed enhanced antiproliferative activities compared to the parental drug, artemisinin. Compound **29b** displayed the most potent anti-cancer activity on PC12 cancer cell lines with an IC_50_ of about 1.56 mM. The antiproliferative mechanism study showed that dimer **29b** inhibits the cell cycle at the G1 phase and stimulated cell apoptosis through up-regulation of Bad, caspase-3, and caspase-9 protein expressions while hindering the expression of Bcl-xL [50]. The length of the linker had a significant impact on the anticancer activity of these compounds. Some of the compounds with shorter chain lengths exhibited reduced anticancer activity and those with longer chain lengths displayed enhanced anticancer activity. Compound **29b** was among the compounds with phosphate moiety and these compounds (with phenyl ester and ethyl ester) exhibited good anticancer activity with **29b** (phenyl ester) as the most potent compound against all the cancer cells [50]. 

**Figure 16:**
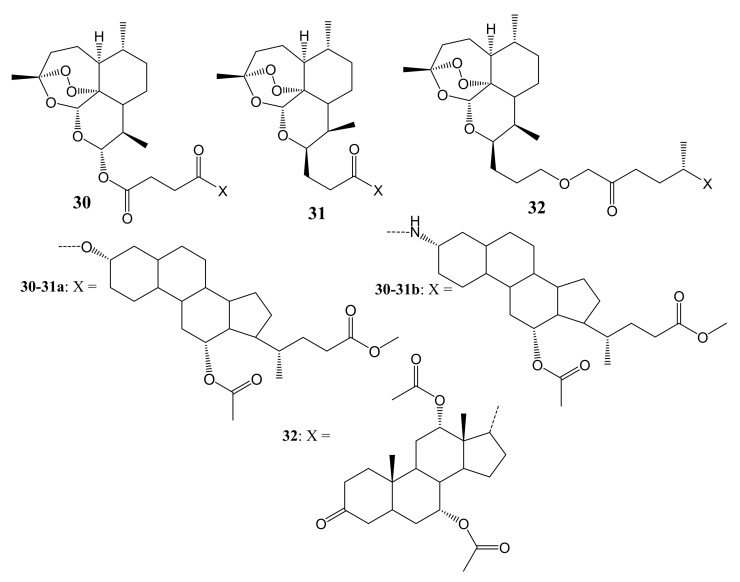
Artesunate-based hybrid molecules incorporated with cholic acid moieties (**30**–**32**).

Letis et al. synthesized artesunate-based hybrid molecules incorporated with cholic acid moieties containing ester and amide linkers for leukemia therapy (Figure 16) [51]. The in-vitro anticancer studies of the hybrid compounds **30**–**32** using sensitive CCRF-CEM and multidrug-resistant CEM/ADR5000 cancer cells showed IC_50_ values that ranged between 0.019 µM and 0.192 µM against CCRF-CEM cancer cells while the IC_50_ values of artemisinin was (36.90 ± 6.90 µM) and artesunic acid (0.07 ± 0.03 µM), suggesting outstanding antileukemia activity of the hybrid compounds. These hybrids also showed interesting high cytotoxicity against multidrug-resistant CEM/ADR5000 cells. Remarkably, the amide compound **31b** was the most potent against CCRF-CEM (IC_50_ = 0.019 ± 0.001 µM) and CEM/ADR5000 (IC_50_ = 0.345 ± 0.031 µM) with relatively low degree of cross-resistance (18.2) [51]. The cytotoxicity comparison from these synthesized compounds showed that position C-3 of the cholic acid moiety is important. The alpha–orientation of either amino group or hydroxyl in that position enhanced the anticancer activity of the hybrids. Another important factor that influenced the activity of these compounds was the ester linkage and two carbon chains between the two moieties (artemisinin and cholic acid) that resulted in superior cytotoxic effects on the CEM/ADR5000 cells [51]. Alpha–orientation at C-3 of the cholic acid moiety for both the amide and ester influenced the cytotoxicity of the compounds.

**Figure 17:**
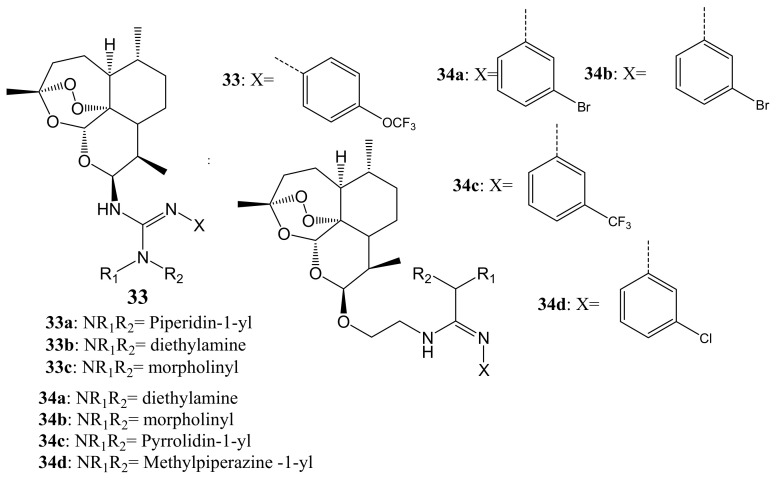
Artemisinin–guanidine hybrids (**33**,**34**).

Xie et al. synthesized artemisinin–guanidine hybrids via aza-Wittig reaction (Figure 17). The in-vitro examination of their anticancer activity against MDA-MB-231, HT-29, and A549 cell lines revealed moderate to excellent activity. All the dimer compounds, except **33a**–**c** and **34d**, demonstrated a 2-to 4-fold more cytotoxic effect against A549 than against MDA-MB-231 cancer cells, which showed their excellent selective inhibition effect against the A549 cancer cell line [52]. Hybrids substituted on the beta-C-10 position of guanidine moiety exhibited a better inhibitory effect than compounds substituted on the alpha-C-10 position of guanidine moiety when tested against A549 cancer cells [52]. The introduction of oxyethyl- flexible linkage between the two moieties was useful for the anticancer activity of these compounds [52].

**Figure 18:**
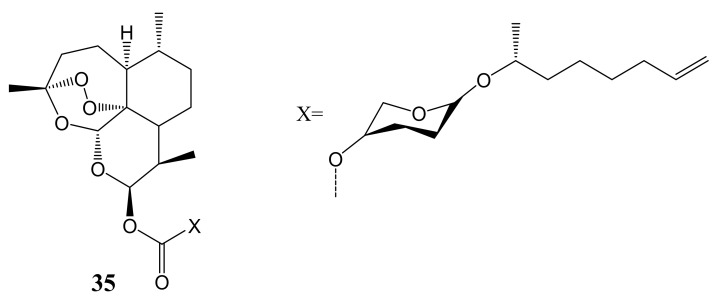
Deoxoartemisinin-glycolipid hybrid (**35**).

Ricci et al. synthesized deoxoartemisinin-glycolipid hybrid, **35** (Figure 18) that demonstrated superior antitumor efficacy against oral carcinoma cancer cell lines with less than 20µM effective concentration when compared to the free artemisinin or glycolipid. It showed five times more anti-oral cancer efficacy than either paclitaxel or cisplatin. Similar compounds (deoxoartemisinin-glycolipid hybrids) were synthesized by Min et al.and they exhibited good antitumor efficacy against MCF-7, A549, and MDA-MB-231 when compared to the parental drugs [53]. The compounds with 12β(C–O)- type of substitution exhibited inferior anticancer activity when compared to compounds with 12β(C–O)- type of substitution. Regiospecificity is one of the paramount factors in the development of potent artemisinin anticancer agents [53].

**Figure 19:**
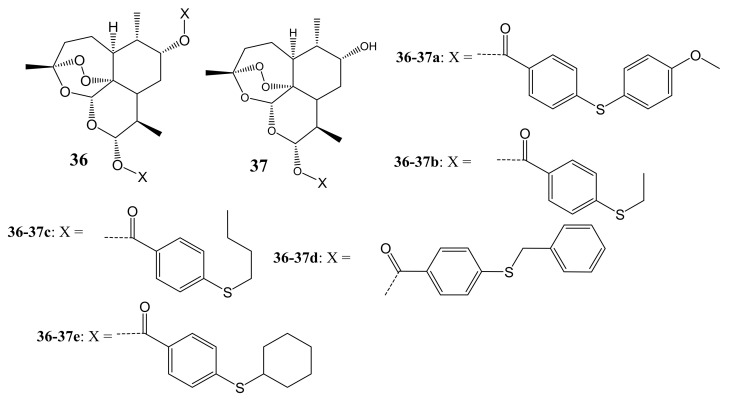
DHA-based hybrid compounds (**36**,**37**).

Xu et al. synthesized DHA-based hybrid compounds bonded with sulfur molecules (Figure 19) for cancer therapy and confirmed their structures using ^13^C NMR, ^1^H NMR, and HRESIMS [54]. The cytotoxicity studies in vitro utilizing MTT assay demonstrated that hybrids **36a** and **37a** possessed superior anticancer activity against PC-3, A549, and SGC-7901 cell lines with IC_50_ values ranging between 1.6 and 30.5 mM, while other hybrid compounds exhibited poor antitumor activity against these cancer cell lines. The structure-activity relationship (SAR) analysis showed no clear trend on the effects of the side chains on the anticancer activity of hybrids **36a** and **37a** [54].

**Figure 20:**
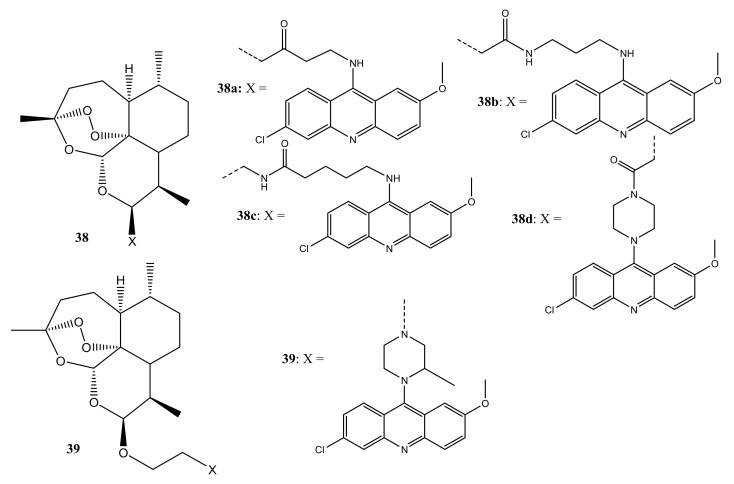
Artemisinin–acridine hybrid compounds (**38**,**39**).

Jones et al. designed and synthesized artemisinin–acridine hybrid compounds (**38a**–**d**) (Figure 20) for the treatment of cancer [55]. The anti-tumour studies showed that compounds **38a**–**d** display favourable cytotoxicity effects against MCF-7, MDA-MB-231, and leukemia (HL60) cancer cells with its cytotoxic effect ranked in the order: MDA-MB-231 < MCF-7 < HL60. Flow cytometry analysis showed that these hybrids promote cell death by apoptosis and bind covalently to their intraparasitic cellular targets in the presence of iron (II). Combining an artemisinin derivative with an acridine demonstrated enhanced anticancer efficacy in the HL60 cancer cell line with **38d** displaying a remarkable cytotoxic effect which was superior to **38a**–**c** with dihydroartemisinin IC_50_ values of 0.56 µM. However, it displayed a higher inhibitory effect against MDA-MB-231 and HT29-AK cell lines [55]. Additionally, these observations showed that the length of the linker between the two moieties had an impact on the anticancer activity of these hybrids as compound **37d** has the longest chain length. Thus, it is essential to consider the length of the linker in the development of novel artemisinin agents [55]. 

Moreover, Joubert et al. reported another library of artemisinin–acridine hybrid compounds (Figure 20) that demonstrated a higher cytotoxic effect against HeLa cell lines when compared with chloroquine and melphalan, indicating that the hybrids can be used for the treatment of cervical cancer [56]. Compound **39** with 2-methyl piperazine as a linker showed potent anticancer activity towards HeLa cell lines and it was 12 and 5-fold higher than melphalan and chloroquine, respectively [56]. These results proved that artemisinin–acridine hybrids with 2-methyl piperazine are potential antitumor agents. Compounds with 2-methyl piperazine linker exhibited remarkable anticancer activity, in vitro [55,56]. 

**Figure 21:**
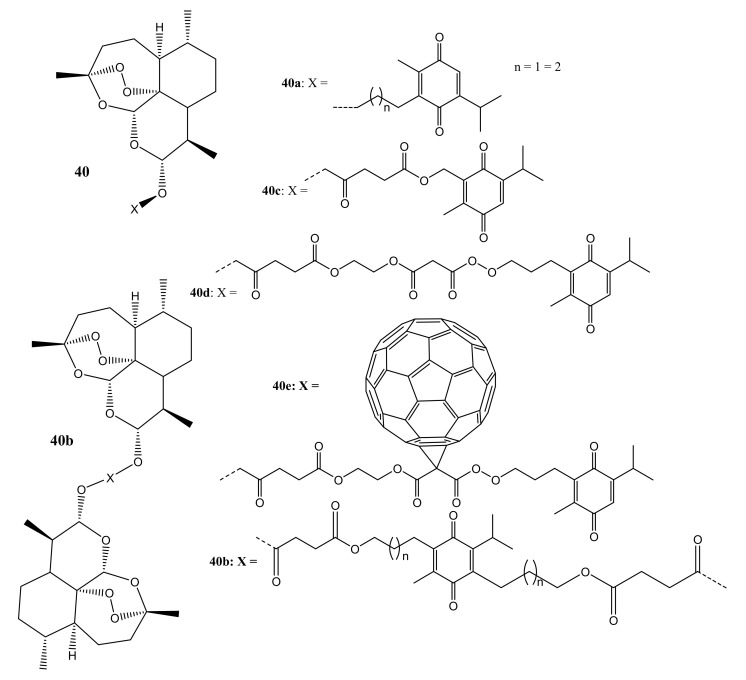
Thymoquinone-artemisinin hybrids (**40a**–**e**).

Frohlich et al. synthesized artemisinin-thymoquinone hybrids (**40a**–**e**) that display antileukemia, antimalarial, and antiviral activity [57]. The hybrid compounds are listed in Figure 21. All hybrid compounds displayed a moderate to good antileukemic activity except compound **40e** (which consist of fullerene moiety) against both the doxorubicin-resistant and doxorubicin sensitive-cell lines with EC_50_ values that range between the micromolar to the submicromolar range (EC_50_ (CCRF-CEM) = 0.0027−6.071 μM; EC_50_ (CEM/ADR5000) = 0.2−5.663 μM), which in all cases were superior when compared to that of parental drug, artemisinin (EC_50_ (CCRF-CEM) = 36.90 μM; EC_50_ (CEM/ADR5000) = 26.90 μM). These results revealed that the length of the linker plays a vital role in the antileukemia efficacy of artemisinin dimers, where thymoquinone is used as a spacer molecule [57].

**Figure 22:**
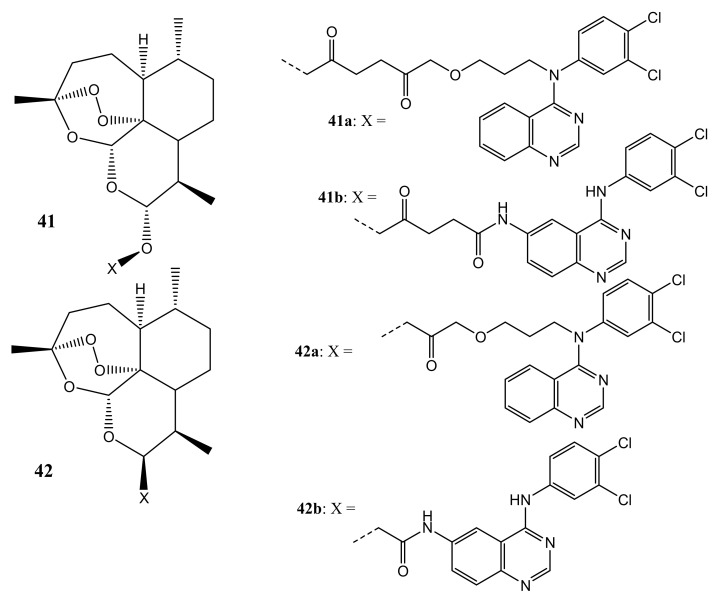
Artemisinin-quinazoline hybrids (**41**,**42**).

Also, Frohlich et al. synthesized hybrids that are based on artemisinin and quinazoline (**41**,**42**) (Figure 22) with antileukemia activity. The most promising compounds were **41b** and **42b,** and they were evaluated in vitro for cytotoxic effect against two leukemia cell lines [58]. Compound **41b** displayed good antileukemia activity against CCRF-CEM and CEM/ADR5000 cells with EC_50_ values of 2.8 μM and 0.5 μM, exhibiting that their antileukemia effects are similar to that of artesunic acid. The efficacy of this compound was more promising against the multidrug-resistant CEM/ADR5000 leukemia cells, as it was 45-fold more active than doxorubicin (EC50 = 23.27 μM), suggesting that it can overcome the emergence of drug resistance in the present cancer therapy. However, hybrid compound **42b** did not display any promising antileukemia effects [58]. The amide bonds present on the linker of these compounds influenced the anticancer activity of these compounds.

**Figure 23:**
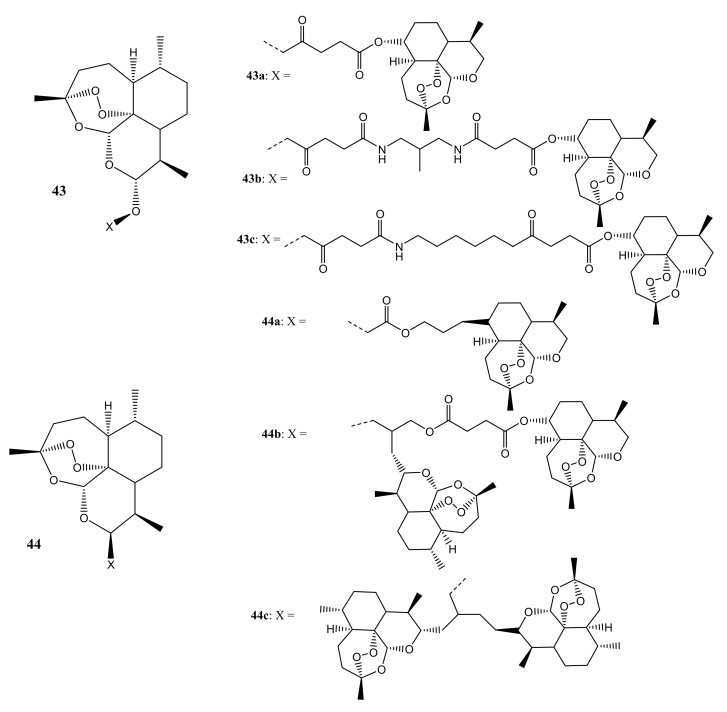
Artemisinin-based dimers and trimmers (**43**,**44**).

Reiter et al. synthesized artemisinin-based dimers and trimmers (**43**,**44**) containing ester, amide, and ether linkers (Figure 23**)** as potential anticancer drugs for the treatment of leukemia [59]. The in vitro anticancer studies demonstrated that hybrid **44a** possessed an IC_50_ of 0.09 µM against CCRF-CEM and IC_50_ of 0.20 µM against CEM/ADR5000 cells, exhibiting similar activity as DHA and artesunic acid. Hybrids **44a**, **44b,** and **44c** (IC_50_ values 60.49 µM) proved to be versatile agents useful against the multidrug-resistant cell line CEM/ADR5000. The compounds were three times as active as doxorubicin (IC_50_ value of 1.61 µM). Regarding the antileukemic activity, it was observed that C-10 non-acetal dimers (e.g., hybrid **44a**) were more potent in vitro when compared to C-10 acetal compounds (e.g., hybrid **43**) [59].

## 4. Artemisinin-Based Hybrid Compounds for Malaria Therapy

### 4.1. Artemisinin-Quinoline Hybrids

**Figure 24:**
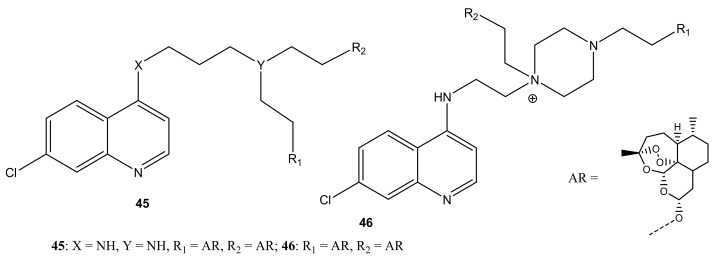

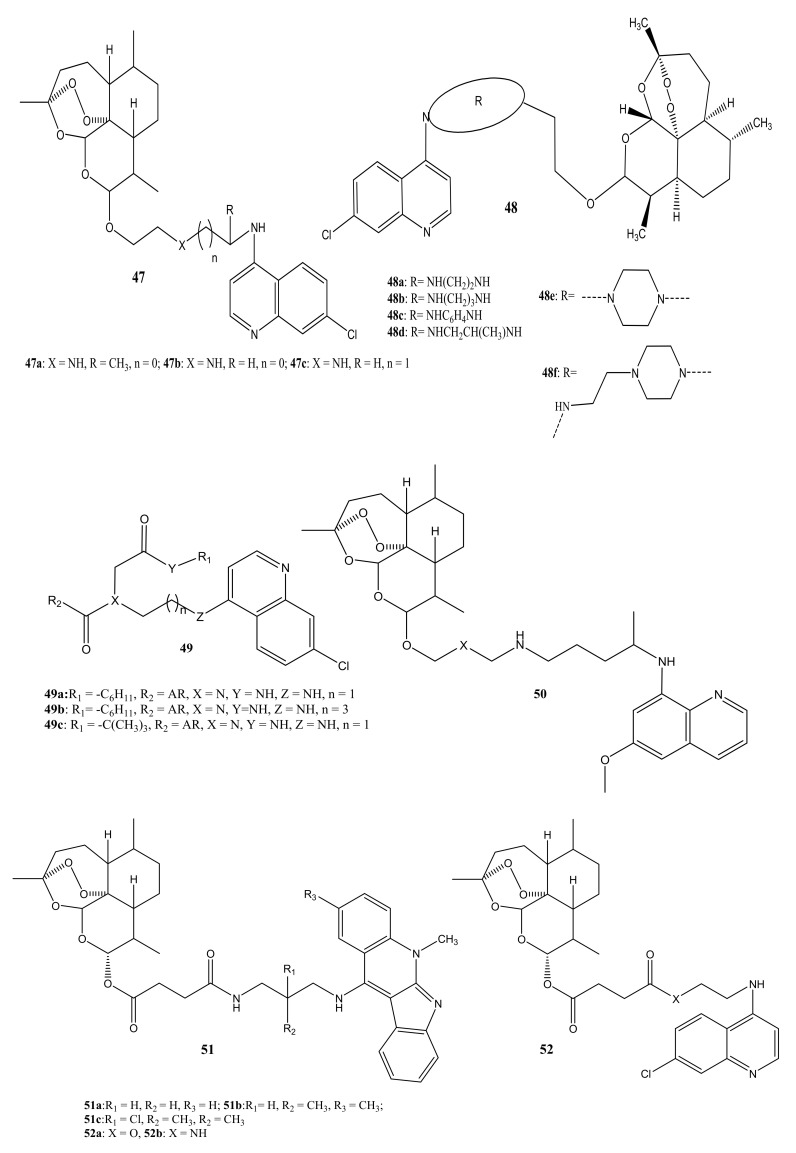

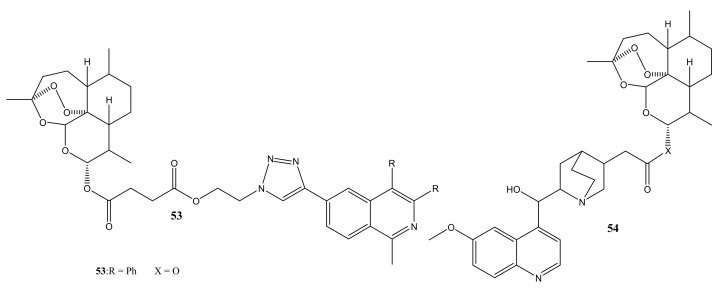
Artemisinin-quinoline hybrid compounds (**45**–**54**).

There are reports of artemisinin-quinoline compounds with potent antimalarial activity (Figure 24). Lombard et al. prepared artemisinin-quinoline compounds (**45**,**46**) and evaluated their in vitro antiplasmodial activity against *P. falciparum* CQ-S 3D7, D10, and CQ-R Dd2 strains, using CQ and dihydroartemisinin (DHA) as controls [60]. The compounds displayed promising antimalarial activity that was equivalent to CQ and DHA against all the strains of *P. falciparum* used in the study. The activity of the compounds was influenced by the length of the linker. Increasing the linker length resulted in a decrease in antimalarial activity [60]. Lombard et al. also prepared and evaluated the in-vitro and in-vivo activity of artemisinin-quinoline hybrid compounds **47** against *P. vinckei*. The compounds displayed effective antimalarial activity against CQ-S and CQ-R strains. The in vivo results revealed a complete parasitemia clearance at 15 mg/kg and 50 mg/kg via intraperitoneal and oral administration, respectively [61]. 

Lombard et al. prepared artemisinin-aminoquinoline hybrid compounds **48a**–**f** and evaluated their antiplasmodial activity in vitro against CQ-S D10 and CQ- R Dd2 strains of *P. falciparum* with CQ and DHA used as the reference drugs. For solubility and stability reasons, oxalic acid was reacted with the hybrid compounds to produce oxalate salts which were also tested in vitro. The compounds displayed moderate activity against Dd2 and D10 with IC_50_ values in the range (12.18 and 201.38 nM) and (17.12–275.99 nM), respectively. Most of the compounds displayed better antimalarial activity than CQ on both strains. The oxalate salts displayed better antimalarial activity than the hybrid compounds [62]. Feng et al. prepared artemisinin-chloroquinoline analogues, **49a**–**c,** and evaluated their antiplasmodial activity in vitro against CQ-S D10 and CQ-R K1 strain with CQ being used as the reference drug. The compounds exhibited good antiplasmodial activity that was similar to CQ (IC_50_ = 20 nM) against D10 (IC_50_ = 25, 27 and 35 nM). The activity of the compounds was better than CQ (219 nM) for K1 (IC_50_ = 19, 21 and 23 nM) [63]. Capela et al. prepared a hybrid compound, **50** containing primaquine and dihydroartemisinin scaffolds. The in vitro and in vivo evaluation of the compounds against *Plasmodium* blood and liver stages showed that the compounds were more active against the liver stages. The in vitro studies revealed that the hybridization of primaquine with DHA increased the efficacy of primaquine [64]. 

Wang et al. prepared artesunate-indoloquinoline hybrid compounds, **51a**–**c,** and the in vitro results showed that the compounds exhibited enhanced antimalarial activity than the reference drugs. The hybrid compounds displayed low cytotoxicity and were good inhibitors of β-hematin formation. In vivo studies in *P. berghei* infected mice by oral or intraperitoneal administration of compound **51a** once in a day in dosed of 10 mg K^−1^ for four consecutive days, reduced the parasitemia on day 4 with an antiparasitic activity of 89.6% and mean survival time of 7.7 days [65]. Lorion et al. prepared artemisinin-quinoline **52** and artemisinin-isoquinoline **53** hybrid compounds. The hybrids were more effective against the resistant strains than the sensitive strains and they showed improved efficacy compared to the parent drugs. In-vivo studies showed that the hybridization via ester linkage improved the efficacy of the individual drugs because compound **52a** displayed greater activity than artesunate in *P. berghei* infected mice [66]. Walsh et al. prepared a hybrid compound containing artemisinin and quinine via an ester linkage. Compound **54** displayed superior antimalarial activity than the parent drugs, suggesting that hybridization enhances their antimalarial activity [67]. 

### 4.2. Ferrocene-Artemisinin Hybrids

The first development of ferrocene-artemisinin hybrid compounds started two decades ago but the study of their antimalarial efficacy (ferroprotoporphyrin IX binding ability) is ongoing [68]. Various researchers have synthesized different ferrocene-artemisinin compounds and tested their antimalarial activity against chloroquine-resistant (CQ-R) and chloroquine-sensitive (CQ-S) strains of *P. falciparum* and their SAR have been discussed [4]. Moreover, the reports from various authors showed that the type of linkers (functional groups, position of the introduction, type of a bond, length of a linker, and type of heterocycles) introduced between ferrocene moiety and artemisinin influenced the therapeutic effects of the synthesized compounds [4]. 

### 4.3. Artemisinin Hybrids Containing Other Antimalarials

**Figure 25:**
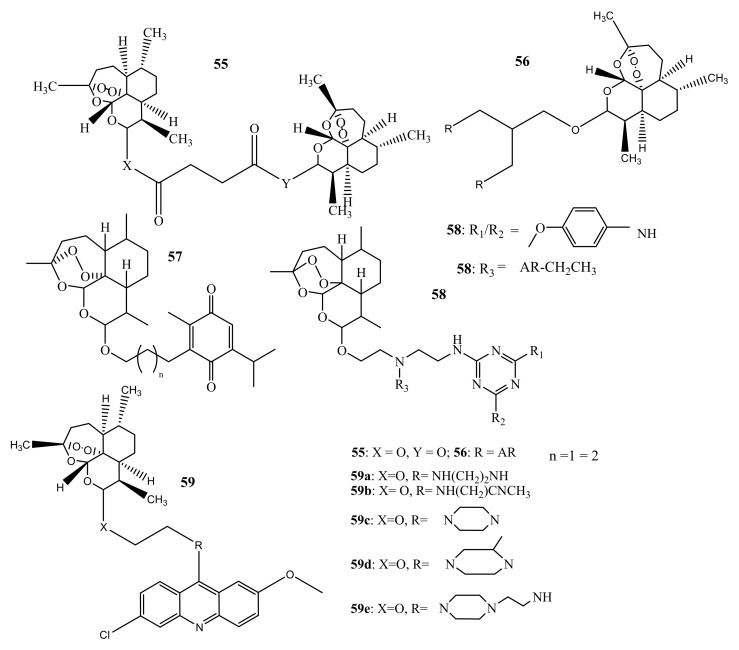
Artemisinin-based hybrid compounds (**55**–**59**).

Reiter et al. prepared dimers and trimers of artemisinin (**55**–**59**) (Figure 25) and tested their in-vitro antiplasmodial activity against CQ-S 3D7 strains of *P. falciparum* with CQ and DHA used as reference drugs. The hybrid compounds displayed promising activity with IC_50_ ranges of 2.6–12.8 nM. The activity of compounds **55** (2.6 nM) and **56** (2.6 nM) was greater than CQ (9.8 nM), but almost the same as DHA (2.5 nM). The activity of the dimers was greater than the trimers and this suggests that increasing peroxides groups does not enhance the antimalarial activity [69]. Frohlich et al. synthesized artemisinin-thymoquinone **57** hybrid compounds via an ether linkage. The compounds displayed promising antimalarial and were more active than CQ. However, the compounds were less active than dihydroartemisinin. No trend could be followed regarding the effect of the length of the linker on the compounds [57]. Cloete et al. prepared artemisinin-triazine hybrids and hybrid dimers, and the compounds showed minimum activity in comparison with DHA and AS against *P. falciparum*-sensitive strains. However, for resistant strains, the compounds were more active than CQ. The hybrid dimers were more active, and the compounds were found to be selectively toxic to parasitic cells in the presence of mammalian cells [69]. Joubert et al. prepared artesunate-acridine hybrid compounds and tested their antimalarial activity in vitro against *P. falciparum* CQ-S NF54 and CQ-R Dd2 strains using CQ as the control. The hybrid compounds displayed promising activity with IC_50_ in the range of 2.6–266.8 nM against NF54 and 35.3–429.9 nM against Dd2 strains. The decreased activity against Dd2 implies that the compounds cannot overcome resistance. Compound **58a** was the most effective compound against both strains and had a shorter linker [56]. 

## 5. Conclusions 

The search for novel, cost-effective and therapeutic agents with improved efficacy for the treatment of cancer and malaria has resulted in the reports of several hybrid compounds containing artemisinin scaffolds. Some of the hybrid compounds displayed significant antimalarial and anticancer activity in vitro and in vivo with the potential to overcome drug resistance with good selectivity and low toxic side effects. In some of the research reports, the length and the nature of the linkers between the parent drugs played a significant role in the biological outcomes of the compounds. The combination of artemisinin and its derivatives with known pharmacophores is a promising strategy for the development of improved and effective drugs. This approach also has the potential to overcome the shortcomings associated with artemisinin and its derivatives such as short half-life, poor solubility, limited bioavailability, etc. Despite all the research reports which have revealed the efficacy of artemisinin and its derivatives, there is still a pressing need to fully understand the mode of actions of these compounds. The continuous development and research on hybrid compounds containing artemisinin and derivatives will result in potent anticancer and antimalarial agents.
